# Laminoplasty versus laminectomy for multi-level cervical spondylotic myelopathy: a systematic review of the literature

**DOI:** 10.1186/1749-799X-8-45

**Published:** 2013-12-01

**Authors:** Lifeng Lao, Guibin Zhong, Xinfeng Li, Lie Qian, Zude Liu

**Affiliations:** 1 Department of Orthopedic Surgery, Renji Hospital, Shanghai Jiaotong University School of Medicine, Shanghai 200127, China

**Keywords:** Laminoplasty, Laminectomy, Skip laminectomy, Cervical spondylotic myelopathy

## Abstract

**Background:**

There is considerable controversy as to which posterior technique is best for the treatment of multi-level cervical spondylotic myelopathy. The aim of this study was to compare the clinical and radiographic results and complications of laminoplasty (LAMP) and laminectomy (LAMT) in the treatment of multi-level cervical spondylotic myelopathy.

**Methods:**

We reviewed and analyzed papers published from January 1966 and June 2013 regarding the comparison of LAMP and LAMT for multi-level cervical spondylotic myelopathy. Statistical comparisons were made when appropriate.

**Results:**

Fifteen studies were included in this systematic review. There was no significant difference in the incidence of surgical complications between LAMP and LAMT. Compared to conventional LAMT and skip LAMT, postoperative ROM was more limited in LAMP, but this was still superior to postoperative ROM following LAMT with fusion. Postoperative kyphosis occurred in 8/180 (4.44%) in LAMP and 13/205 (6.34%) in LAMT, whereas no cases of kyphosis were reported for skip LAMT. Skip LAMT appears to have better clinical outcomes than LAMP, while the outcome was similar between LAMP and LAMT with fusion.

**Conclusions:**

Based on these results, a claim of superiority for laminoplasty or laminectomy was not justified. In deciding between the two procedures, the risks of surgical and neurological complications, and radiologic and clinical outcome, must be taken into consideration if both options are available in multi-level cervical spondylotic myelopathy.

## Introduction

Cervical spondylotic myelopathy is a progressive disease that often requires surgical intervention [1]. A variety of surgical options exist, including anterior and posterior approaches, which may or may not involve fusion. Cervical spondylotic myelopathy surgery is often multi-level, which can complicate the surgical management. Even when discussion is limited to posterior procedures, there is considerable controversy as to which technique is best for multi-level posterior cervical decompression. The oldest posterior approach is laminectomy (LAMT), which can be performed with or without fusion [2]. Recently, a modification has been introduced, called skip LAMT [3]. Laminoplasty (LAMP) techniques were developed to avoid complications of LAMT such as segmental instability and postlaminectomy kyphosis in 1982 [4].

Consultation between surgeons is inadequate for establishing clinical equipoise between two alternative treatment options for the management of a specific disease [5]. Despite ongoing uncertainty regarding the most effective surgical methods for posterior approach for multi-level cervical spondylotic myelopathy, few systematic reviews have explored this issue. A randomized, controlled trial is therefore necessary to determine the best currently available treatment for multi-level cervical spondylotic myelopathy.

To address this, this study aimed to perform a systematic review of LAMP and LAMT for the treatment of multi-level cervical spondylotic myelopathy, specifically evaluating their clinical and radiographic results and complications, as an aid to guide clinical decision-making and provide information which may be useful in the design of a randomized controlled trial.

## Materials and methods

### Research questions

Three clinically relevant research questions, based on safety and efficacy, and designed to address the goal of defining the optimal surgical treatment for multi-level cervical spondylotic myelopathy patients, were determined by consensus of a panel of spine surgeons: Question 1: *Given a multi-level cervical spondylotic myelopathy that could be treated with either LAMP or LAMT, which treatment would be optimal with regard to complications?*; question 2: *In patients with multi-level cervical spondylotic myelopathy treated with either LAMP or LAMT, which treatment is good in radiographic outcomes?*; question 3:: *Which is superior to the other in clinical outcomes in patients with multi-level cervical spondylotic myelopathy treated with either LAMP or LAMT?* Clinical (radiographic) and/or safety data were the primary evidentiary outcomes use to answer each question.

### Selection criteria

The studies were selected based on the following inclusion criteria: addressing the population of interest (adult patients with multi-level cervical spondylotic myelopathy), type of study (clinical studies), types of interventions (LAMP compared with LMPT in treatment of multi-level cervical spondylotic myelopathy), and outcome measures (based on complications, radiographic outcome, or patient-related outcome measures with regard to pain and quality of life using various validated questionnaires, e.g., Japanese Orthopedic Association scores and Nurick scores). Case reports, studies describing novel or unconventional techniques (e.g., endoscopic decompression), and clinical studies with less than 1-year follow-up were excluded.

### Identification of studies

Publications comparing LAMP and LAMT in the management of multi-level cervical spondylotic myelopathy were identified from a Medline search between January 1966 and June 2013 using the OVID search engine with “laminoplasty”, “laminectomy”, and “cervical spondylotic myelopathy” or “cervical spondylosis” or “cervical myelopathy” as keywords and with MeSH (Medline/PubMed's article indexing terminology) subject headings. Two authors reviewed the titles, and if the title suggested any possibility that the article might meet eligibility criteria, the abstracts were retrieved and reviewed. The authors then chose potentially eligible studies for retrieval. The review of complete articles for eligibility included only the methods section and was thus blinded with regard to author, institution, journal, and results. Data on the outcomes listed above were extracted by two reviewers, and any differences were resolved by discussion. This study was approved by the Ethics Committee of Renji Hospital.

### Statistical analysis

Unpaired *t* test and chi-squared test were used for statistical analysis. Fisher's exact test was used when the expected values in any of the cells of a contingency table are below 5. A *P* value of <0.05 was considered statistically significant.

## Results

Fifteen studies comparing LAMP and LAMT in treating multi-level cervical spondylotic myelopathy were included in this systematic review [6-20]. The characteristics of the included studies are summarized in Table 1. Among the studies, LAMP surgical procedures mainly refer to open-door laminoplasty with different fixation, while LAMT surgical procedures were divided into three subgroups as follows: conventional LAMT [6-8,11,13,18], skip LAMT [10,12,14], and LAMT with fusion [9,15-17,19,20].

**Table 1:** Data of publication of the management of laminoplasty versus laminectomy for multi-level cervical compressive myelopathy

**Reference**	**Study design**	**Year, journal**	**Patients**	**Age**	**Characteristics of patients**	**Procedure**	**Follow-up**	**Surgical complications**	**Clinical outcome**	**Radiographic outcome**	**Fusion rates**	**Hardware failure**
			**(no.)**	**(year)**								
[6]	Retrospective	1988, *Spine*	LAMP 15	LAMP 64	Multi-level cervical spondylosis	Open-door LAMP;	>2 years	LAMP: subluxation 8, closing of the open door 2	LAMP: 86% patients were excellent or good	ROM was more limited in LAMP	N/A	N/A
						complete LAMT with bilateral partial facetectomy						
			LAMT 12	LAMT 64.2				LAMT: kyphosis 3, subluxation 9	LAMT: 66% patients were excellent or good, *P* < 0.05			
[7]	Retrospective	1988, *Spine*	LAMP 75	LAMP 55	Cervical spondylotic myeloradiculopathy, OPLL	Open-door LAMP;	LAMP 10.8 years	N/A	JOA score improvement: LAMP 81.4%	N/A	N/A	N/A
						LAMT without damage to the facets						
			LAMT 14	LAMT 59.2			LAMT 4.6 years		LAMT 81.1%			
[8]	Retrospective	1988, *J Bone Joint Surg Br*	LAMP 18	N/A	N/A	French window LAMP;	>5 years	LAMP: kyphosis 5, instability 5	JOA score:	Limitation of extension was more remarkable after LAMP	N/A	N/A
									No significant difference			
			LAMT 10			LAMT		LAMT: kyphosis 3, instability 3				
[9]	Retrospective	2001, *Spine*	LAMP 13	LAMP 56	Multi-level cervical myelopathy	Open-door/T-saw LAMP;	LAMP 26.2 months (12–46 months)	LAMP 0	Nurick score: a greater percentage of patients in LAMP group reported a subjective improvement	Significantly greater reduction sagittal plane motion in LAMT	LAMT 61.5% (8/13)	LAMT 2
								LAMT: myelopathy progression 2, subjacent degeneration 1, infection 1, kyphosis 1, graft site pain 2, revision surgery 1				
			LAMT 13	LAMT 55		complete LAMT and fusion						
							LAMT 25.5 months (9–62 months)					
									*P* > 0.05	*P* < 0.01		
[10]	Retrospective	2003, *Spine*	LAMP 51	LAMP 67	Multi-level cervical spondylosis, OPLL, spinal canal stenosis	Open-door LAMP; skip LAMT	LAMP 43 months (24–66 m)	LAMP: C5 paresis 3	Average recovery rates: *P* > 0.05	Recovery rate of ROM: LAMP 44%, LAMT 98%, *P* < 0.05	N/A	N/A
									Axial symptoms: LAMP 66.7% (34/51), LAMT 2% (1/43), *P* < 0.05			
			LAMT 43	LAMT 69								
							LAMT 30 months (24–41 months)	LAMT: laminar fracture 3, CSF leakage 2	Difficulty in looking around: LAMP 76% (39/51), LAMT 0% (0/43)			
[11]	Retrospective	2004, *Iowa Orthop J*	LAMP 20	LAMP 53.5	Multi-level cervical spondylotic myelopathy or radiculopathy	Open-door LAMP with rib allograft;	LAMP 65.4 months (36–112 months)	LAMP: C5 paresis 2, closure of the open door 1	Modified Nurick scale improvement: LAMP: 43.6%, LAMT 17.8%, *P* < 0.0001	ROM: LAMP 27° in extension, LAMT 43° in extension, *P* < 0.001	N/A	N/A
			LAMT 22	LAMT 54.3		LAMT						
							LAMT 64.8 months (53–76 months)	LAMT: wound dehiscence 1, subluxation 2, kyphosis 3				
									VAS score improvement: LAMP 57%, LAMT 8%, *P* < 0.01			
[12]	Prospective	2007, *Spine*	LAMP 21	LAMP 62.3	Cervical myelopathy and spinal cord compression	Double-door LAMP;	28.1 months (12–48 months)	No complications in the two groups	Recovery rate of JOA score, *P* > 0.05	Recovery rate of ROM: LAMP 77.4%, LAMT 88.6%, *P* > 0.05, C2–C7 lordosis, *P* > 0.05	N/A	N/A
						Skip LAMT						
			LAMT 20	LAMT 66.1					VAS score: *P* > 0.05, supplemental analgesic demands: *P* > 0.05			
[13]	Retrospective	2010, *Neurol Res*	LAMP 72	LAMP 59.7	Cervical spondylotic myelopathy or radiculopathy	LAMP;	4 months	LAMP: infection 1, wound dehiscence1	LAMP had better result in Rankin score, Glasgow outcome score, and Karnofsky score (*P* < 0.01)	N/A	N/A	N/A
						Complete LAMT with preserving the facet joints						
			LAMT 49	LAMT 57.3				LAMT: infection2, wound dehiscence1				
									Nurick score: *P* > 0.05			
[14]	Prospective	2010, *J Spinal Disord Tech*	LAMP 25	LAMP 62.4	Cervical spondylotic myelopathy and spinal cord compression	Double-door LAMP;	>2 years	LAMP: infection 1	SF12 scores for physical and mental health: *P* > 0.05	Recovery rate of ROM: LAMP 46%, LAMT 84%, *P* < 0.05	N/A	N/A
						Skip LAMT						
			LAMT 25	LAMT 69.6				LAMT: infection 1	SF12 scores for cervical pain: better for LAMT, *P* < 0.05			
[15]	Retrospective	2011, *Clin Orthop Relat Res*	LAMP 39	LAMP 60	Multi-level cervical spondylotic myelopathy	LAMP using Mitek suture anchor fixation;	Average of 24 months	LAMP: chronic pain 2, recurrent stenosis 1, persistent radiculopathy 1, revision surgery 2	Gait or pain postoperatively: *P* > 0.05	Sagittal alignment postoperatively: better in LAMP, *P* < 0.05	LAMT: 98.8% (81/82)	LAMT 1
			LAMT 82	LAMT 64					Neck pain:			
						LAMT and fusion			P > 0.05			
								LAMT: chronic pain 2, dysphagia 1, infection 1, junctional stenosis 1, kyphosis 1, revision surgery 2		Junctional kyphosis: *P* > 0.05		
[16]	Retrospective	2011, *J Neurosurg Spine*	LAMP 30	LAMP 61	Cervical stenotic myelopathy	Instrumented, open-door LAMP; LAMT and fusion	LAMP 42.3 months (13–69 months)	LAMP: infection 2, sterile seromas 2, C5 paresis 1, urinary retention 1, revision surgery 4	Nurick score or JOA score: *P* > 0.05	Radiographic outcomes were similar between the groups	LAMT 92% (24/26)	LAMP 2
												LAMT 2
			LAMT: 26	LAMT: 58					VAS score improvement: LAMP -0.2 (pain scores increased slightly postoperatively)			
							LAMT: 41.3 m (12-85m)					
								LAMT: infection 4, sterile seromas 2, C5 paresis 1, revision surgery 7	LAMT 2.8, *P* < 0.05			
[17]	Prospective	2012, *Neurosurgery*	LAMP 9	LAMP 61	Multi-level cervical spondylotic myelopathy with or without radiculopathy	Open-door expansile LAMP;	>12 months	No complications in the two groups	Nurick grade, SF-36 score, Neck disability index, self-reported outcome measures were improved only in LAMP, *P* < 0.05	ROM was decreased only in LAMT, *P* < 0.05	N/A	N/A
			LAMT 7	LAMT 55		LAMT and fusion				Percent of change in area of spinal canal: LAMP 34%, LAMT 76%, *P* < 0.01		
[18]	Retrospective	2012, *Neurosurgery*	LAMP 154	LAMP 67	Cervical radiculopathy or myelopathy	Standard LAMP; LAMT	LAMP 96 months	N/A	LAMP was associated with more neck pain and worse quality of life (4 or more levels involved); there was no difference (3 or fewer levels)	A greater extent of decompression in LAMP, P < 0.05	N/A	N/A
			LAMT 114	LAMT 73			LAMT 58 months					
										Sagittal alignment: *P* > 0.05		
									VAS score: *P* > 0.05			
									EQ-5D questionnaire: improve significantly in LAMT			
[19]	Retrospective	2013, *Eur Spine J*	LAMP 36	LAMP 57.1	Multi-level cervical degenerative myelopathy	Open-door LAMP; LAMT and fusion	LAMP 9.2 months (7–11 months)	N/A	Final follow-up JOA score and neurological recovery rate: *P* > 0.05	Loss of curvature index: LAMP 2.60 ± 1.01, LAMT 1.22 ± 0.72, *P* < 0.05	N/A	N/A
			LAMT 32	LAMT 55.9								
									Axial symptom incidence: LAMP 66.7 % (24/36), LAMT 37.5 % (12/32), *P* < 0.05			
							LAMT 8.9 months (7–12 months)					
[20]	Retrospective	2013, *Orthopedics*	LAMP 75	LAMP 57.2	Multi-level cervical stenotic myelopathy	Plate-only open-door LAMP;	>24 months	LAMP: C5 paresis 3, CSF leakage 1, kyphosis 3, restenosis 1, axial pain 9	JOA score and Nurick score: *P* > 0.05	Increase of dural sac area: LAMP 31.9%, LAMT 52.7%, P < 0.001	LAMP 98.67% (74/75)	N/A
			LAMT 66	LAMT 57		LAMT and fusion			NDI scores and VAS scores: better improvement in LAMP, *P* < 0.05			
										Spinal cord shift: LAMP 1.2 mm, LAMT 2.4 mm, *P* < 0.001	LAMT 96.97% (64/66)	
								LAMT: C5 paresis 11, CSF leakage 3, kyphosis 2, infection 1, axial pain 23	Better neck function recovery in LAMP			
										Curvature index: *P* > 0.05	*P* > 0.05	
										Greater loss of ROM in LAMT		

*LAMP* laminoplasty, *LAMT* laminectomy, *ROM* range of motion, *OPLL* ossification of the posterior longitudinal ligament, *N/A* not available, *CSF* cerebrospinal fluid.

### Operative time and estimated blood loss

Seven studies reported operative time and estimated blood loss of LAMP and LAMT procedures (Table 2). Of these, five reported that the operative time for LAMP was shorter than that for LAMT. Across all seven studies reporting operative time, the LAMP procedures took an average of 137.4 min (*n* = 276), compared to 142.6 min in the LAMT procedures (*n* = 197). Among these same studies, the average blood loss was 299.6 ml in the LAMP patients (*n* = 276) compared to 225.0 ml in LAMT 197 patients. However, two of the seven studies reported that the estimated blood loss for LAMP was less than that for LAMT; interestingly, the LAMT procedure used in these two studies included fusion [17,20].

**Table 2:** Operative time and blood loss of LAMP versus LAMT for cervical spondylotic myelopathy

**Reference**	**Year**	**Operative time of LAMP (min)**	**Operative time of LAMT (min)**	**Blood loss of LAMP (ml)**	**Blood loss of LAMT (ml)**
[7]	1988	151	169.2	505	343.3
[10]	2003	114	133	249	18
[11]	2004	201	165	505	310
[12]	2007	63	77	44	43
[14]	2010	108	70	105	50
[17]	2012	180	210	405	500
[20]	2013	145.1	173.8	284.5	310.9

### Surgical complications

Across six studies [6,8,9,11,15,20], kyphosis was found in 8 of 180 (4.44%) patients treated with LAMP and 13 of 205 (6.34%) patients treated with LAMT. There was no significant difference between the two techniques in kyphosis incident (*P* > 0.05). In four studies [10,11,16,20], C5 paresis was found in 9 of 176 (5.11%) patients treated with LAMP and 12 of 157 (7.64%) patients treated with LAMT. Across six studies [9,13-16,20], infection was found in 4 of 254 (1.57%) patients treated with LAMP and 10 of 261 (3.83%) patients treated with LAMT. There was no significant difference between the two techniques in the incidence of kyphosis, C5 paresis, or infection (*P* > 0.05).

Other surgical complications that occurred in LAMP and LAMT include subluxation, instability, cerebrospinal fluid leakage, wound dehiscence, urinary retention, chronic pain, restenosis, nonunion, hardware failure, and revision surgery. Again, there was no significant difference between the two techniques in the incidence of these complications (*P* > 0.05) (Table 3).

**Table 3:** Comparison of surgical complications after LAMP or LAMT

**Complication**	**References**	**LAMP (%)**	**LAMT (%)**	***P *** **value**
Kyphosis	[6,8,9,11,15,20]	8/180 (4.44)	13/205 (6.34)	0.413
C5 paresis	[10,11,16,20]	9/176 (5.11)	12/157 (7.64)	0.343
Infection	[9,13-16,20]	4/254 (1.57)	10/261 (3.83)	0.192
Subluxation	[6,11]	8/35 (22.86)	11/34 (32.35)	0.377
Instability	[8]	5/18 (27.78)	3/10 (30.05)	1.000
CSF leakage	[10,20]	1/126 (0.79)	5/109 (4.59)	0.154
Wound dehiscence	[11,13]	1/92 (1.09)	2/71 (2.82)	0.820
Urinary retention	[16]	1/30 (3.33)	2/26 (7.69)	0.899
Chronic pain	[15,20]	11/114 (9.65)	25/148 (16.89)	0.091
Restenosis	[15,20]	2/114 (1.75)	1/82 (1.22)	1.000
Nonunion	[9,15,16,20]	6/157 (3.82)	5/187 (2.67)	0.547
Hardware failure	[9,15,16]	2/82 (2.44)	5/121 (4.13)	0.797
Revision surgery	[9,15,16]	6/82 (7.32)	10/121 (8.26)	0.806

In addition, clothing of the open door was found in 3 of 35 (8.57%) patients [6,11], persistent radiculopathy in 1 of 39 (2.56%) patients [15], and sterile seromas in 2 of 30 (6.67%) patients [16], treated with LAMP. Myelopathy progression of the open door was found in 2 of 13 (15.38%) patients [9], subjacent degeneration in 1 of 13 (7.69%) patients [9], graft site pain in 2 of 13 patients (15.38%) [9], laminar fracture in 3 of 43 (6.98%) patients [10], and dysphagia in 1 of 82 (1.22%) patients [15], treated with LAMT.

### Radiographic outcome

Four studies reported radiographic outcome after LAMP or standard LAMT [6,8,11,18]. Compared to standard LAMT, three studies reported that postoperative range of motion (ROM) was more limited in LAMP (*P* < 0.05) [6,8,11]. While, Nurboja et al. reported that sagittal alignment (Ishihara Index) was similar in the two groups [18]. Interestingly, the radiological effectiveness of decompression was greater in the LAMP group (*P* < 0.05).

Three studies reported radiographic outcome after LAMP or skip LAMT [10,12,14]. In all of these, the mean percentage postoperative ROM was better in skip LAMT, but this was statistically significant in only two studies [10,14].

Six studies reported radiographic outcome after LAMP or LAMT with fusion [9,15-17,19,20]. Five of these studies found a greater loss of ROM and more of an increase of dural sac area in LAMT with fusion, compared to LAMP (*P* < 0.05). However, Highsmith et al. reported that the radiographic outcomes were similar between the two groups and that the patients in both groups lost 3°–4° of lordosis but maintained a lordotic curve (*P* > 0.05) [16].

### Clinical outcome

Although there was no uniform criterion for the assessment, all 15 studies reported clinical outcome of LAMP and LAMT. The clinical outcome was evaluated according to Odom's criteria, Japanese Orthopedic Association scores, Nurick scores, Visual Analogue Scale (VAS) score, Rankin score, Karnofsky score, Glasgow outcome score, SF-36 score, SF12 score, EQ-5D questionnaire, neurological recovery rate, and patients' self-assessment. Of these 15 studies, 5 reported that the clinical outcome of LAMP was similar to that of LAMT [7-9,12,15]; another 5 reported that the clinical outcome of LAMP was better than of LAMT [6,11,13,17,20]; and the remaining 5 studies reported that the clinical outcome of LAMP was worse than that of LAMT [10,14,16,18,19]. Of the three studies comparing LAMP and skip LAMT, skip LAMT had a better clinical outcome than LAMP in two studies [10,14], while remained similar in one study [12]. Of the six studies comparing LAMP and LAMT with fusion, the clinical outcome of LAMT with fusion was better in two studies [16,19], worse in two studies [17,20], and similar in another two studies [9,15].

### Economic analysis

Only one study performed an economic comparison between LAMP and LAMT with fusion procedures. The hardware costs of a C3-6 construct were US$4,200 for LAMP with no allograft versus US$12,000 for LAMT with a mini-polyaxial fusion construct of the same length (without crosslink). Implant costs in LAMT with fusion cases were nearly triple those of LAMP cases. Even after correcting for the longer constructs used in the LAMT with fusion cases, the implants were still over twice as costly. Most of the fusion complications occurred when the fusion extended to T-1 or below. Crossing the cervicothoracic junction increased hardware requirements and the risk of reoperation, thus raising costs considerably [16].

## Discussion

Surgical treatment of multi-level cervical spondylotic myelopathy remains controversial and challenging. LAMP and LAMT are two of the most commonly performed posterior procedures for the treatment of multi-level cervical spondylotic myelopathy. However, it is unclear whether multi-level cervical spondylotic myelopathy is best treated with LAMP, LAMT, skip LAMT, or LAMT with fusion. The aim of this study was to document operative time, blood loss, surgical complications, radiographic outcome, and clinical outcomes of LAMP and LAMT for multi-level cervical spondylotic myelopathy, so as to help surgeons to compare these two options.

Comparative studies against LAMT have demonstrated the safety and efficacy of the LAMP procedure. Yonenobu et al. reported a direct comparison of LAMP to subtotal corpectomies and fusion for the treatment of multi-level cervical spondylotic myelopathy [21]. Their retrospective single-institution comparison demonstrated that although the two procedures have similar rates of functional recovery, LAMP is associated with a lower rate of complications. In 1988, Herkowitz compared anterior cervical fusion, LAMT, and LAMP for the management of multi-level spondylotic radiculopathy [6]. In his retrospective review of 45 patients, a successful outcome was found in 86% and 66% of patients undergoing LAMP and LAMT, respectively. A noteworthy distinction in complication rates was observed with the anterior procedure (70%) faring worst, followed by LAMT (25%) and LAMP (13%). Other limitations typically reported for LAMP include a 30%–50% decrease in cervical sagittal motion and postoperative axial discomfort in a high percentage of patients [22]. Our review found that kyphosis occurred in 8/180 (4.44%) patients undergoing LAMP and 13/205 (6.34%) of patients undergoing LAMT. Interestingly, we found no reported cases of kyphosis when skip LAMT was used. Nonunion, hardware failure, and revision surgery occurred mainly in LAMP and LAMT with fusion, with again no reported cases of these in skip LAMT.

In 2004, Kaminsky et al. compared LAMP and standard LAMT without fusion in a case control study [11]. The Nurick scores of the patients in the LAMP group improved by a mean of 0.96, with those patients having fewer complications than patients in the LAMT (without fusion) group, whose scores improved by a mean of 0.59. In addition, Kaminsky et al. [11] found fewer late complications in the LAMP group compared to LAMT.

Skip LAMT is a recently developed minimally invasive procedure. In a comparative study, Shiraishi et al. [10] reported that only 1 patient (2%) undergoing skip LAMT had newly developed axial pain, whereas 33 patients (66%) treated with LAMP had postoperative development or deterioration of axial pain. The atrophy rate of the deep extensor muscles in skip LAMT averaged 13%, whereas that in LAMP was 59.9%. In the LAMP group, three patients (5.7%) had C5 paresis, while none occurred in the skip LAMT group. Skip LAMT also had better postoperative ROM, relative to LAMP (*P* <0.05). Skip LAMT was found to be less invasive to posterior extensor structures, including the deep extensor muscles, than LAMP. Additionally, skip LAMT was effective in preventing postoperative morbidities, often seen after conventional LAMT and LAMP with adequate decompression of the spinal cord. Sivaraman et al. [14] also reported less blood loss, short operative times, significantly improved axial pain scores, and significantly improved preservation of range of movement with skip LAMP, compared to LAMT. The degrees of decompression with both techniques were similar. However, Yukawa et al. [12] reported that no significant differences were seen between skip LAMT and LAMP, in terms of operative invasiveness, axial neck pain, cervical alignment, ROM, and clinical results.

There is ample evidence from biomechanical experiments [23,24], suggesting that lateral mass screws could provide rigid fixation to the multiple cervical planes: flexion stability increased 92%, extension stability increased 60%, and rotation stability improved greatly. Yang et al. [20] reported that LAMT with fusion can achieve a greater extent of enlargement of the spinal canal and spinal cord drift compared with LAMP. However, the degree of neurological functional recovery was similar in the LAMT with fusion and LAMP groups, while neck function was worse in the LAMT with fusion group. Axial symptoms are strongly correlated with cervical ROM [20]. LAMT with fusion achieves intervertebral stability at the expense of losing a greater ROM, which may cause stiffness and muscle atrophy. Heller et al. [9] compared the results of LAMT with fusion against LAMP and noted an almost twofold decrease in the postoperative ROM in the LAMT with fusion group. The LAMP with fusion group also suffered from significantly more complications, leading Heller et al. to conclude that LAMP might be preferred to LAMT with fusion as a posterior procedure in patients with cervical spondylotic myelopathy.

In terms of clinical and radiographic outcome evaluation, score index methods were commonly applied, including JOA score, Nurick score, VAS score, Rankin score, Karnofsky score, Glasgow outcome score, SF-36 score, SF12 Score, EQ-5D questionnaire and percentage ROM, Ishihara index, and curvature index. There was therefore no uniform criterion for assessment, and inconsistent result is sometimes reported even within the same study. Hardman et al. [13] reported LAMP had better result in Rankin score, Glasgow outcome score, and Karnofsky score than conventional LAMT (*P* < 0.01), but no significant difference in Nurick scores (*P* > 0.05). Highsmith et al. [16] reported that the Nurick and JOA scores were similar (*P* > 0.05) between LAMP and LAMT with fusion, but the VAS score was wore in the LAMP group (*P* < 0.05). Du et al. [19] reported that the final follow-up JOA score and neurological recovery rate were similar between LAMP and LAMT with fusion (*P* > 0.05), but axial symptom incidence was much higher in the LAMP group (66.7%) compared with LAMT (37.5%) (*P* < 0.05). Yang et al. [20] also reported that JOA and Nurick scores were similar between LAMP and LAMT with fusion (*P* > 0.05) but found that the NDI and VAS scores were more improved with LAMP (*P* < 0.05).

There are some limitations in this systematic review. Incomplete searching of the literature is one potential limitation; however, the use of MEDLINE, PubMed, EMBASE, and the Cochrane Database suggests that all of the most important articles addressing this issue were discovered. We only assessed articles in English; therefore, articles written in other languages are likely to have been missed. The second limitation was that surgical procedure was not always uniform, with studies making use of LAMT, skip LAMT, or LAMT with fusion. In addition, some studies included in this systematic review involved procedures performed without accompanying instrumentation. Most studies included in this systematic review were retrospective, and only three studies were prospective [12,14,17], approaches which are likely to give differing indications of LAMP and LAMT performance. To be able to draw a more reliable conclusion about the management of multi-level cervical spondylotic myelopathy, further randomized, controlled prospective studies should be designed in the future.

In conclusion, there was no significant difference between the two techniques in operative time, estimated blood loss, and surgical complications. Compared to standard LAMT and skip LAMT, postoperative ROM was more limited in LAMP, yet LAMT with fusion resulted in the greatest limitation of ROM. The clinical outcome evaluation results included in this review were not uniform. Skip LAMT seemed to have better clinical outcome than LAMP, while the outcome was similar between LAMP and LAMT with fusion. Based on these results, a claim of superiority for LAMP or LAMT was not justified. In deciding between the two procedures, the risks of surgical and neurological complications, and radiologic and clinical outcome must be taken into consideration if both options are available in multi-level cervical spondylotic myelopathy.

## Competing interests

The authors declare that they have no competing interests.

## Authors' contributions

The design of the study and preparation of the manuscript were done by LL and ZL. LL and XL assisted in the study processes, data collections, and preparations. GZ and LQ assisted in the manuscript preparation. All authors read and approved the final manuscript.
